# Age Variation Among US Adults’ Social Media Experiences and Beliefs About Who Is Responsible for Reducing Health-Related Falsehoods: Secondary Analysis of a National Survey

**DOI:** 10.2196/56761

**Published:** 2024-11-27

**Authors:** Prathyusha Galinkala, Elise Atkinson, Celeste Campos-Castillo

**Affiliations:** 1 Michigan State University, Media and Information, 404 Wilson Rd, East Lansing, MI, 48824, United States, 1 8183887141

**Keywords:** social media, health misinformation, gray digital divide, United States, older adults, aging, health information, false information, falsehoods

## Abstract

**Background:**

We live in a digital age where social media has become an essential part of people’s lives. It is also one of the leading platforms responsible for spreading health-related falsehoods. This study explores who adults of different age groups perceive as responsible for reducing health-related falsehoods on social media.

**Objective:**

Despite growing concern over older adults’ exposure to false health information on social media, little research examines their beliefs on how to address the problem. This study examines how the age of US adults is associated with their reported experiences with health-related falsehoods on social media and their beliefs about who should be tasked with reducing such falsehoods.

**Methods:**

This study is a secondary analysis of data from the 2022 Health Information National Trends Survey, a nationally representative survey of US adults (18 years and older). Multivariable logistic regressions estimated how a respondent’s age was associated with their self-reported social media use, their difficulty to detect health-related falsehoods on social media, their discussion of health information found on social media with medical providers, and their beliefs regarding who should be responsible for reducing health-related falsehoods on social media. Regression estimates were adjusted for respondents’ sociodemographic and health characteristics.

**Results:**

Daily social media use decreased with respondents’ age. Respondents aged 50‐64 years (*b*=0.515, *P*=.01) and 65‐74 years (*b*=0.697, *P*=.002) were more likely than respondents aged 18‐34 years to report they strongly agree that it is difficult for them to detect health-related falsehoods on social media. Compared to younger adults, older adults (65‐74 years: *b*=0.818, *P*=.002; 75 years and older: *b*=1.058, *P*<.001) were more likely to believe medical providers should be responsible for reducing online falsehoods.

**Conclusions:**

In addition to ongoing efforts by social media platforms to detect and remove falsehoods, the findings suggest medical providers should be tasked with discrediting health-related falsehoods on social media for older adults. However, time during the clinical visit is limited. Future research is needed to discover new approaches and tools tailored to older adults to assist with filtering and discrediting health-related falsehoods on social media.

## Introduction

The COVID-19 pandemic heightened attention to concerns about the prevalence of false health-related information circulating on social media [1,2]. Of particular concern is exposure to falsehoods among older adults (≥65 years old), who generally have less experience with social media than younger adults [3]. Older adults are more inclined to interact with false information [4,5], perhaps due to their longer experience managing truths from both digital and nondigital news sources [6]. Compared to younger adults, older adults exhibit less confidence in discerning false information [7]. Altogether, this suggests older adults are more susceptible to harms stemming from health-related falsehoods on social media.

To reduce online falsehoods, various entities have adopted several measures, including the use of algorithms by social media companies to automatically detect falsehoods, but these are neither built nor deployed with older adults in mind [8,9]. Few studies examine the perspectives of older adults regarding their beliefs about the best ways to address falsehoods online. An exception is a Pew Research Center survey from 2021, in which older adults were the least likely to prefer social media platforms address online falsehoods [10]. However, it is unknown what older adults would prefer, especially given the COVID-19 pandemic potentially altering their adoption and use of information and communication technologies. To address this gap, we analyzed data collected in 2022 from a national sample of US adults to understand how respondents’ age is associated with their social media experiences and their beliefs about the best way to address health-related falsehoods online. We discuss how the findings could be used to shape the policy-making process and to design interventions to assist older adults in filtering out falsehoods.

## Methods

### Sample

We derived data from the 2022 Health Information National Trends Survey (HINTS), a cross-sectional survey by the National Cancer Institute to document how noninstitutionalized US adults (≥18 years old) access and process health information. Complete survey design details are available elsewhere [11]. Briefly, the survey was fielded from March to November 2022 and included 6242 respondents, with a final weighted response rate of 28.1%.

The outcomes of interest, which broadly assessed experiences with and beliefs about falsehoods on social media, were asked only of respondents who reported social media use (n=4415). Among these respondents, most missing values were for the item asking respondents their race and ethnicity (n=439), which we accounted for by including an indicator for those with a missing response. Respondents with missing values on other measures were dropped from the analysis. The final analytic sample comprised 3941 respondents.

### Measures

We analyzed three survey items to characterize respondents’ experiences with social media and falsehoods. Daily social media users were identified as those who reported they visited a social media site “Almost every day.” Self-reported difficulty to detect health-related falsehoods was measured using their level of agreement with the item, “I find it hard to tell whether health information on social media is true or false.” Discussion of social media with medical providers was determined based on their level of agreement with the item, “I use information from social media in discussions with my health care provider.” The latter two items were ordinal variables with four response options each. Because the proportional odds assumption was violated, to simplify the table summaries, we present results in the main text that compare those selecting the modal response option (assessment: strongly agree; discussion: strongly disagree) to all others (assessment: somewhat agree, somewhat disagree, strongly disagree; discussion: somewhat disagree, somewhat agree, strongly agree). Multimedia Appendix 1 includes full results using partial proportional odds models to analyze the outcomes in their original ordinal form. Conclusions about older adults from the simplified models shown in the text are the same as those drawn from analyses that address the violation via estimating partial proportional odds models.

A fourth item determined respondents’ beliefs regarding who should reduce health-related falsehoods on social media. The question posed was, “Who do you think has the main responsibility for reducing the amount of false or misleading health information on social media?” The five response options included news media, social media platforms, government, individual users, and medical providers. The respondents were limited to select only one option.

Respondents’ age was measured using categories (18‐34, 35‐49, 50‐64, 65‐74, and ≥75 years). We adjusted estimates based on respondents’ sociodemographic and health background. These were measures of respondents’ sex (male, female), race and ethnicity (Black, Latino, White, other, missing), education (high school or less, some college, college graduate, and postgraduate), relationship status (single, divorced/widowed/separated, and married/cohabitating), and self-rated health (excellent, very good, good, fair, poor).

### Statistical Analyses

After characterizing the sample by examining frequencies and proportions, we estimated eight binary logit regressions to understand how respondents’ age was associated with the outcomes of interest. The first three logit regressions estimated responses to items assessing participants’ experiences with social media and health-related falsehoods. Because self-reported difficulty to detect falsehoods and discussion of social media information with providers may be less likely among more frequent social media users, we included being a daily social media user as a covariate for these regressions. The next five regressions estimated responses to the item assessing participants’ beliefs about who should be responsible for reducing health-related falsehoods on social media. The item had five possible options, and thus each regression estimated the likelihood of selecting a given option. Because one possible option was social media companies, we included being a daily social media user as a covariate for these regressions. All regressions adjusted estimates based on covariates. All analyses were weighted (except for the calculation of frequencies) with survey weights provided by the HINTS methodologists to approximate the US adult population, which accounted for the sampling design and nonresponses. Analyses were performed with Stata 18. Statistical significance is defined as *P*<.05, determined by a 2-tailed *t* test.

### Ethical Considerations

Ethics review for this study was waived because it is a secondary analysis of deidentified survey data.

## Results

### Sample Characteristics

Table 1 summarizes sample characteristics. Approximately 14.7% of the sample comprised older adults (≥65 years old). The majority of the sample (73.1%) reported daily usage of social media. Around 58.3% of the respondents reported they strongly disagree that they discuss social media with their health care provider, and around 28.8% of the respondents reported they strongly agree that it is difficult for them to detect health-related falsehoods on social media. When considering responsibility for addressing health-related falsehoods on social media, approximately 13.1% of the sample believed the news media should take responsibility, 34% felt social media platforms were responsible, around 15.5% assigned the responsibility to the government, 22.5% believed individual users of social media should take action, and 14.8% believed it should be medical providers.

**Table 1.** Sample characteristics.

Characteristic	Frequency, n (weighted %)^a^	
Respondents reported they strongly agree that it is difficult for them to detect health-related falsehoods on social media	1338 (28.8)	
Respondents reported they use social media daily	2771 (73.1)	
Respondents reported they strongly disagree that they discuss social media information with health care practitioners	2312 (58.3)	
Who should reduce falsehoods		
News media	473 (13.1)	
Social media platforms	1452 (34)	
Government	578 (15.5)	
Individual users	843 (22.5)	
Medical providers	595 (14.8)	
Sex		
Female	2474 (53.2)	
Male	2176 (46.8)	
Age (years)		
18‐34	747 (29)	
35‐49	966 (29)	
50‐64	1181 (27.3)	
65‐74	728 (10)	
≥75	319 (4.7)	
Education		
High school or less	782 (25.1)	
Some college	1157 (39.6)	
College graduate	1175 (20.3)	
Postgraduate	827 (15)	
Race and ethnicity		
White	2183 (59.3)	
Black	603 (11.1)	
Latino	704 (17.3)	
Other	348 (10.5)	
Missing	103 (1.8)	
Self-rated health is excellent, very good, or good	3295 (84.4)	
Relationship status		
Single	821 (32.4)	
Divorced, widowed, or separated	951 (10.1)	
Married or cohabitating	2169 (57.5)	

a Weighted frequencies were calculated using the weighted averages provided by the Health Information National Trends Survey.

### Logit Regressions

The binary logit regressions estimating how respondents’ age was associated with their experiences with social media and health-related falsehoods are shown in Table 2. Older adults were significantly less likely to use social media daily than 18‐34 year olds (65‐74 year olds: *b*=−1.603, *P*<.001; ≥75 year olds: *b*=−1.427, *P*<.001). Adults aged 35‐49 years were significantly more likely than those aged 50 to ≥75 years to strongly disagree they discuss social media with their health care provider (*b*=0.309, *P*=.04). Adults aged 50-74 years old were significantly more likely than 18‐34 year olds to strongly agree that it is difficult for them to detect health-related falsehoods on social media (50‐64 years olds: *b*=0.515, *P*=.01; 65‐74 years olds: *b*=0.697, *P*=.002).

**Table 2.** Binary logit regression predicting experiences with social media and falsehoods online.

	Respondents who reported they use social media daily			Respondents who reported they strongly disagree that they discuss social media information with health care practitioners			Respondents who reported they strongly agree that it is difficult for them to detect health-related falsehoods on social media		
Variable	*b^a^*	SE	*P* value	*b*	SE	*P* value	*b*	SE	*P* value
Female (vs male)	0.283	0.128	.03	−0.070	0.116	.55	0.198	0.154	.20
Age (years; vs 18‐34 years)									
35‐49	−0.953	0.223	<.001	0.309	0.149	.04	0.140	0.148	.35
50‐64	−1.106	0.213	<.001	−0.100	0.150	.51	0.515	0.194	.01
65‐74	−1.603	0.274	<.001	0.118	0.138	.40	0.697	0.211	.002
≥75	−1.427	0.262	<.001	−0.011	0.205	.96	0.422	0.235	.08
Race and ethnicity (vs White)									
Black	−0.342	0.192	.08	0.042	0.174	.81	−0.264	0.186	.16
Latino	0.170	0.198	.40	−0.287	0.152	.06	−0.289	0.147	.054
Other	−0.194	0.189	.31	−0.257	0.187	.18	−0.308	0.196	.12
Missing	−0.636	0.321	.053	0.438	0.291	.14	0.633	0.375	.10
Education (vs high school or less)									
Some college	0.299	0.166	.08	−0.038	0.157	.81	0.358	0.161	.03
College graduate	0.189	0.177	.29	−0.189	0.157	.23	0.383	0.166	.82
Postgraduate	−0.074	0.181	.68	−0.243	0.176	.17	−0.418	0.195	.04
Relationship status (vs single)									
Divorced, widowed, or separated	−0.037	0.165	.82	0.269	0.170	.12	0.203	0.221	.36
Married or cohabitating	0.083	0.154	.56	0.129	0.155	.41	0.028	0.175	.87
Daily social media use	—^b^	—	—	−0.331	0.124	.01	−0.243	0.138	.86
Self-rated health is excellent, very good, and good	0.088	0.149	.56	−0.024	0.181	.90	0.234	0.160	.15

a *b*: sample regression coefficient.

b As being a daily social media user was a covariate and the regression was between daily social media users against daily social media users, this did not generate any values.

Table 2 also shows that, compared to respondents with high school or less education, postgraduates were less likely to strongly agree that it is difficult to detect health-related falsehoods on social media (*b*=−0.418, *P*=.04), while those with some college education were more likely to agree (*b*=0.358, *P*=.03). This pattern does not hold in the supplemental analyses in Multimedia Appendix 1.

Table 3 summarizes binary logit regressions estimating how respondents’ age was associated with their beliefs about who should be responsible for reducing health-related falsehoods on social media. Respondents aged 75 years and older were significantly less likely than 18‐34 year olds to believe individual users are responsible for reducing online falsehoods (*b*=−0.888, *P*<.001). Compared to 18‐34 year olds, older adults were significantly more likely to believe medical providers were responsible (65‐74 year olds: *b*=0.818, *P*=.002; ≥75 year olds: *b*=1.058, *P*<.001). Respondents’ ages were unrelated to the likelihood of selecting any of the other three entities (news media, social media platforms, government) as responsible.

**Table 3.** Binary logit regressions predicting beliefs about who should reduce falsehoods online.

	News media			Social media platforms			Government			Individual users			Medical providers		
Variable	*b^a^*	SE	*P* value	*b*	SE	*P* value	*b*	SE	*P* value	*b*	SE	*P* value	*b*	SE	*P* value
Female (vs male)	0.079	–0.19	.68	0.083	–0.117	.48	–0.109	–0.183	.55	–0.254	–0.139	.07	0.253	–0.173	.15
Age (years; vs 18‐34)															
35‐49	–0.188	–0.29	.52	0.079	–0.178	.66	–0.102	–0.204	.62	0.1	–0.183	.59	–0.044	–0.259	.87
50‐64	–0.061	–0.263	.82	–0.012	–0.187	.95	0.078	–0.216	.72	–0.251	–0.185	.18	0.355	–0.198	.08
65‐74	–0.405	–0.273	.14	0.25	–0.195	.21	–0.492	–0.266	.07	–0.464	–0.235	.054	0.818	–0.254	.002
≥75	0.084	–0.307	.79	–0.138	–0.208	.51	–0.008	–0.341	.98	–0.888	–0.888	<.001	1.058	–0.223	<.001
Race and ethnicity (vs White)															
Black	–0.004	–0.252	.99	–0.229	–0.153	.14	0.52	–0.267	.06	–0.213	–0.208	.31	0.12	–0.24	.62
Latino	0.08	–0.222	.72	–0.134	–0.165	.42	0.607	–0.243	.02	–0.161	–0.196	.42	–0.332	–0.253	.20
Other	–0.196	–0.24	.42	–0.332	–0.214	.13	0.5	–0.254	.054	–0.007	–0.27	.98	0.224	–0.524	.67
Missing	–0.926	–0.475	.06	–0.136	–0.338	.69	0.845	–0.413	.046	0.255	–0.576	.66	–0.363	–0.43	.40
Education (versus high school or less)															
Some college	–0.315	–0.256	.22	–0.32	–0.136	.02	0.072	–0.2	.72	0.575	–0.2	.006	0.107	–0.225	.64
College graduate	–0.67	–0.232	.006	–0.035	–0.152	.82	–0.045	–0.204	.83	0.65	–0.222	.005	–0.103	–0.233	.66
Post graduate	–0.766	–0.27	.007	0.675	–0.186	.72	–0.081	–0.223	.72	0.687	–0.186	.001	–0.275	–0.204	.18
Relationship status (versus single)															
Divorced, widowed, or separated	–0.113	–0.269	.68	0.205	–0.166	.22	–0.037	–0.21	.09	0.164	–0.207	.43	–0.457	–0.247	.07
Married or cohabitating	–0.087	–0.241	.72	0.497	–0.14	.72	–0.083	–0.195	.67	0.264	–0.171	.13	–0.292	–0.172	.10
Daily social media user	–0.254	–0.185	.18	0.145	–0.129	.27	0.131	–0.17	.44	–0.09	–0.147	.54	–0.025	–0.185	.90
Self-rated health is excellent, very good, and good	0.144	–0.202	.48	0.114	–0.16	.48	–0.383	–0.224	.09	0.184	–0.204	.37	–0.108	–0.19	.57

a *b*: sample regression coefficient.

Table 3 shows additional demographic patterns that were unrelated to our focus on age, but warrant detailing. Among these include educational differences. Specifically, college graduates (*b*=−0.670, *P*=.006) and postgraduates (*b*=−0.766, *P*=.007) were less likely than those with high school degree or less to feel that news media should be held responsible. Individuals reporting some college (*b*=0.575, *P*=.006), college graduate (*b*=0.650, *P*=.005), and postgraduate (*b*=0.687, *P*=.001), were more likely than people with high school or less education to believe that individual users should be held responsible for reducing health-related falsehoods online.

## Discussion

From the results, we see that adults aged 50 years or older reported that they strongly agree that it is difficult for them to detect health-related falsehoods on social media. Compared to younger adults (18-34 years old), older adults (≥65 years old) were more likely to believe medical providers should be responsible for reducing online falsehoods. Despite heightened scrutiny of the veracity of health information on social media during the COVID-19 pandemic, little research has examined the public’s perspective on who should be held responsible for reducing the prevalence of falsehoods. In particular, such information would be useful from older adults because, compared to younger adults, they are both more likely to engage with falsehoods on social media and are less confident in deciphering its veracity [1,4-7,9]. We discuss implications stemming from our findings regarding the development of interventions to assist older adults with interpreting the health-related information they find on social media.

Consistent with other research [8,12], we found an age divide for who is likely to use social media. We found older adults were less likely to report using social media daily than younger adults. While the COVID-19 pandemic may have encouraged older adults to turn to social media for pandemic-related information [13-15], the age divide remains among US adults in 2022. This may be due to continued barriers faced among older adults to learn new information and communication technologies [15].

Despite the age divide in social media use, we found that some experiences with health-related information on social media were comparable between older and younger adults. We found older adults were just as likely as younger adults (18‐34 years old) to discuss information they found on social media with health care providers. Overall, only 58% of the respondents reported they strongly disagree that they discuss health information found on social media with medical providers, likely due to concerns about feeling embarrassed or receiving disapproval from the provider [16]. Where we found differences was in the reported difficulty in detecting falsehoods. Specifically, older adults were more likely than younger adults to report they strongly agree that it is difficult for them to detect health-related falsehoods on social media. This is concerning, especially given other research showing older adults are the most likely to be exposed to falsehoods online [5]. Thus, although older adults are less frequent users of social media than younger adults, they tend to be exposed to more falsehoods and are also less confident in their ability to detect them. Such a concentration of falsehood exposure and difficulty detecting falsehoods warrants seeking interventions tailored to this population.

To begin to identify how to tailor interventions, we turn to our final set of findings. Regarding beliefs in who should be responsible for reducing health-related falsehoods on social media, we found older adults were more likely than younger adults to choose medical providers. The differences may be due to older adults exhibiting higher levels of trust in medical providers [17]. Older adults are also more likely to turn to medical providers for health information than their younger counterparts [18]. We also found adults who were aged 75 years and older were less likely than younger adults to believe individual users should be responsible for reducing online falsehoods. This may be due to age differences in confidence in detecting falsehoods, as well as the differences in digital and health literacy skills necessary to curate one’s own social media experience [19]. We found no other associations between the respondents’ age and the selection of any of the other three entities (news media, government, social media platforms). Altogether, these patterns suggest that, while the emphasis within the United States has been for social media platforms to reduce health-related falsehoods, medical providers may be key for designing interventions to assist older adults in identifying and debunking falsehoods.

Besides age, we found additional demographic variables were related to the outcomes studied. Results show that respondents with postgraduate, college graduate, and some college education are more likely than respondents with high school or less education to feel that individual users should be held responsible for reducing health-related falsehoods on social media. This could be in part because individuals who pursue higher education are more likely to exhibit prosocial behavior, which can be understood as an individual’s responsibility to contribute to the social good [20]. We also see that college graduates and individuals with postgraduate education are less likely to feel that news media should be held responsible for removing health-related falsehoods online than respondents with a high school education or less. This could be due to people with a higher level of educational reporting they support freedom of the press, even if that means falsehoods can be published [21].

This study has a few limitations. Because this is a secondary analysis of survey data, and we are limited in the questions available, we cannot evaluate the mechanisms underlying why older adults prefer medical providers as being responsible for reducing falsehoods online. The questions of interest were only asked of those who are current social media users, and thus we cannot tell what the preferences are among those who do not currently use social media but may use it in the future. The analysis also relies on self-reported data, which may be subject to biases. Because the data are cross-sectional, we cannot distinguish age effects from cohort effects. Age effects refer to how a person’s age can biologically influence their physical, cognitive, emotional, and social experiences, while cohort effects refer to how the era in which a group of individuals is born can influence their experiences, behaviors, and attitudes. From the results, we see that adults aged 50‐64 years and 65‐74 years were more likely than younger adults to report they strongly agree that it is difficult for them to detect health-related falsehoods on social media. This finding could be explained by an age effect, perhaps due to age-related cognitive and physical challenges, or a cohort effect because older adults are from a generation that grew up with no internet and no social media [22]. Lastly, the survey was only available in English and Spanish, thereby missing the preferences of those who speak other languages.

Despite these limitations, this study’s findings shed light on targeted interventions for assisting older adults in filtering falsehoods from the health-related information they encounter on social media. Social media platforms have sought to combat falsehoods by implementing algorithms, moderation teams, and options for users to report posts, but falsehoods persist. Only 58% of respondents reported discussing what they found on social media with their medical providers, suggesting an opportunity to spend more time with the small percentage of older adults on social media to discuss the health-related information they encounter. However, time during the clinic visit is limited. Other research has explored how medical providers can present themselves on social media to appear credible and combat falsehoods by sharing their own advice [23], but this still requires time on behalf of the provider. New technological tools may be needed [24], such as a mobile app endorsed by medical providers for older adults to confirm the accuracy of the health information they encounter online. Future research is needed to identify how best to build on our findings to develop and test targeted interventions for older adults that identify and discredit online falsehoods.

## Supplementary material

10.2196/56761 Multimedia Appendix 1 Partial proportional odds model results.

## Abbreviations

HINTS: Health Information National Trends Survey
