# Supplementary material for: Age Variation Among US Adults’ Social Media Experiences and Beliefs About Who Is Responsible for Reducing Health-Related Falsehoods: Secondary Analysis of a National Survey
Source: JMIR Aging. 2024 Nov 27;7:e56761. doi: 10.2196/56761 (PMC11612527; doi:10.2196/56761)
Supplement: Multimedia Appendix 1 [file aging-v7-e56761-s001.docx]

| Appendix Table 1 - Social Media Use | | | | | | | | | |
| --- | --- | --- | --- | --- | --- | --- | --- | --- | --- |
| Variable | Cutpoint 1 | | | Cutpoint 2 | | | Cutpoint 3 | | |
|  | b | s.e. | p | b | s.e. | p | b | s.e. | p |
| Age (vs. 18-34) |  |  |  |  |  |  |  |  |  |
| 35-49 | -1.285 | (.573) | 0.029 | -0.874 | (.364) | 0.020 | -0.952 | (.220) | 0.000 |
| 50-64 | -1.490 | (.490) | 0.004 | -1.059 | (.334) | 0.003 | -1.096 | (.214) | 0.000 |
| 65-74 | -1.789 | (.603) | 0.005 | -1.436 | (.327) | 0.000 | -1.607 | (.271) | 0.000 |
| 75+ | -1.944 | (.532) | 0.001 | -1.609 | (.362) | 0.000 | -1.416 | (.259) | 0.000 |
| Race and ethnicity (vs. White) |  |  |  |  |  |  |  |  |  |
| Black | -0.758 | (.488) | 0.127 | -0.357 | (.264) | 0.182 | -0.356 | (.196) | 0.075 |
| Latino | 0.046 | (.320) | 0.886 | -0.014 | (.281) | 0.961 | 0.160 | (.199) | 0.425 |
| Other | -0.756 | (.446) | 0.096 | -0.084 | (.273) | 0.761 | -0.210 | (.200) | 0.297 |
| Missing | -0.519 | (.560) | 0.359 | -0.278 | (.328) | 0.402 | -0.671 | (.328) | 0.046 |
| Female (vs. Male) | 0.595 | (.252) | 0.022 | 0.583 | (.170) | 0.001 | 0.279 | (.126) | 0.032 |
| Education (vs. high school or less) |  |  |  |  |  |  |  |  |  |
| Some college | 0.091 | (.324) | 0.779 | 0.379 | (202) | 0.067 | 0.297 | (.164) | 0.076 |
| College graduate | 0.207 | (.421) | 0.625 | 0.462 | (.231) | 0.051 | 0.178 | (.175) | 0.314 |
| Post graduate | -0.066 | (.444) | 0.882 | 0.277 | (.251) | 0.276 | -0.093 | (.178) | 0.603 |
| Relationship status (vs. Single) |  |  |  |  |  |  |  |  |  |
| Divorced, widowed, or separated | -0.382 | (.416) | 0.363 | -0.221 | (.269) | 0.415 | -0.068 | (.161) | 0.675 |
| Married or cohabitating | -0.248 | (.390) | 0.529 | -0.002 | (.255) | 0.994 | 0.074 | (.152) | 0.627 |
| Self-rated health is excellent, very good, and good | 0.843 | (.285) | 0.005 | 0.300 | (.191) | 0.121 | 0.081 | (.151) | 0.595 |

| Appendix Table 2 - Discuss with health care provider | | | | | | | | | |
| --- | --- | --- | --- | --- | --- | --- | --- | --- | --- |
| Variable | Cutpoint 1 | | | Cutpoint 2 | | | Cutpoint 3 | | |
|  | b | s.e. | p | b | s.e. | p | b | s.e. | p |
| Age (vs. 18-34) |  |  |  |  |  |  |  |  |  |
| 35-49 | -0.315 | (.147) | 0.038 | -0.221 | (.209) | 0.296 | -0.890 | (.480) | 0.070 |
| 50-64 | -0.996 | (.149) | 0.506 | 0.036 | (.169) | 0.834 | -0.146 | (.518) | 0.780 |
| 65-74 | -0.119 | (.139) | 0.395 | 0.281 | (.161) | 0.087 | -0.151 | (.506) | 0.766 |
| 75+ | -0.007 | (.203) | 0.973 | 0.218 | (.229) | 0.345 | -0.370 | (.817) | 0.653 |
| Race and ethnicity (vs. White) |  |  |  |  |  |  |  |  |  |
| Black | -0.033 | (.175) | 0.850 | 0.012 | (.140) | 0.930 | 1.410 | (.361) | 0.000 |
| Latino | 0.288 | (.152) | 0.064 | 0.187 | (.157) | 0.239 | 0.594 | (.565) | 0.298 |
| Other | 0.238 | (.183) | 0.200 | 0.512 | (.312) | 0.107 | 0.818 | (.589) | 0.171 |
| Missing | -0.404 | (.297) | 0.180 | -0.566 | (.398) | 0.161 | 0.308 | (.910) | 0.737 |
| Female (vs. Male) | 0.619 | (.116) | 0.596 | 0.083 | (.154) | 0.592 | 0.200 | (.408) | 0.625 |
| Education (vs. high school or less) |  |  |  |  |  |  |  |  |  |
| Some college | 0.224 | (.156) | 0.887 | 0.183 | (.197) | 0.358 | -0.174 | (.480) | 0.718 |
| College graduate | 0.189 | (.157) | 0.233 | 0.181 | (.177) | 0.311 | 0.006 | (.498) | 0.991 |
| Post graduate | 0.232 | (.175) | 0.191 | 0.053 | (.196) | 0.789 | -0.916 | (.620) | 0.146 |
| Relationship status (vs. Single) |  |  |  |  |  |  |  |  |  |
| Divorced, widowed, or separated | -0.236 | (.164) | 0.158 | -0.336 | (.227) | 0.144 | -0.382 | (.470) | 0.421 |
| Married or cohabitating | -0.102 | (.150) | 0.501 | -0.285 | (.223) | 0.208 | -0.082 | (.462) | 0.860 |
| Daily social media user | 0.332 | (.122) | 0.009 | 0.262 | (.190) | 0.174 | 0.385 | (.501) | 0.445 |
| Self-rated health is excellent, very good, and good | 0.305 | (.180) | 0.866 | -0.121 | (.203) | 0.556 | -0.711 | (.468) | 0.135 |

| Appendix Table 3 - Hard to tell what is true/false | | | | | | | | | |
| --- | --- | --- | --- | --- | --- | --- | --- | --- | --- |
| Variable | Cutpoint 1 | | | Cutpoint 2 | | | Cutpoint 3 | | |
|  | b | s.e. | p | b | s.e. | p | b | s.e. | p |
| Age (vs. 18-34) |  |  |  |  |  |  |  |  |  |
| 35-49 | -0.329 | (.188) | 0.086 | -0.209 | (.145) | 0.157 | 0.149 | (.156) | 0.347 |
| 50-64 | 0.478 | (.188) | 0.014 | 0.168 | (.201) | 0.406 | 0.500 | (.204) | 0.018 |
| 65-74 | 0.568 | (.254) | 0.030 | 0.540 | (.220) | 0.017 | 0.713 | (.217) | 0.002 |
| 75+ | 0.611 | (.408) | 0.141 | 0.427 | (.293) | 0.151 | 0.467 | (.244) | 0.062 |
| Race and ethnicity (vs. White) |  |  |  |  |  |  |  |  |  |
| Black | 0.267 | (.194) | 0.175 | -0.245 | (.174) | 0.165 | -0.246 | (.180) | 0.177 |
| Latino | -0.282 | (.216) | 0.199 | -0.440 | (.143) | 0.003 | -0.267 | (.149) | 0.079 |
| Other | -0.046 | (.288) | 0.875 | 0.165 | (.207) | 0.430 | -0.317 | (.197) | 0.113 |
| Missing | -0.495 | (.518) | 0.344 | -0.359 | (.333) | 0.286 | 0.634 | (.348) | 0.074 |
| Female (vs. Male) | 0.047 | (.164) | 0.774 | -0.027 | (.117) | 0.822 | 0.205 | (.152) | 0.184 |
| Education (vs. high school or less) |  |  |  |  |  |  |  |  |  |
| Some college | 0.074 | (.207) | 0.722 | 0.169 | (.146) | 0.251 | 0.347 | (.156) | 0.031 |
| College graduate | 0.104 | (.251) | 0.680 | -0.072 | (.163) | 0.660 | 0.003 | (.165) | 0.987 |
| Post graduate | -0.387 | (.245) | 0.120 | -0.504 | (.168) | 0.004 | -0.440 | (.183) | 0.020 |
| Relationship status (vs. Single) |  |  |  |  |  |  |  |  |  |
| Divorced, widowed, or separated | 0.342 | (.213) | 0.114 | 0.053 | (.202) | 0.792 | 0.150 | (.222) | 0.501 |
| Married or cohabitating | -0.731 | (.183) | 0.691 | 0.024 | (.136) | 0.859 | 0.021 | (.180 | 0.909 |
| Daily social media user | 0.302 | (.170) | 0.081 | 0.044 | (.129) | 0.732 | -0.020 | (.135 | 0.882 |
| Self-rated health is excellent, very good, and good | 0.375 | (.226) | 0.102 | 0.723 | (.160) | 0.654 | 0.21 | (.162) | 0.200 |
